# Nutritional supplements and infection in the elderly: why do the findings conflict?

**DOI:** 10.1186/1475-2891-5-30

**Published:** 2006-11-23

**Authors:** Saul Sternberg, Seth Roberts

**Affiliations:** 1 Department of Psychology, University of Pennsylvania, Philadelphia, PA 19104 - 6228, USA; 2 Department of Psychology, University of California, Berkeley, CA, 94720 - 1650, USA

## Abstract

**Background:**

Most of the randomized placebo-controlled trials that have examined the clinical effects of multivitamin-mineral supplements on infection in the elderly have shown no significant effect. The exceptions are three such trials, all using a supplement with the same composition, and all claiming dramatic benefits: a frequently cited study published in 1992, which reported a 50% reduction in the number of days of infection (NDI), and two 2002 replication studies. Questions have been raised about the 1992 report; a second report in 2001 based on the same trial, but describing effects of the supplement on cognitive functions, has been retracted by *Nutrition*. The primary purpose of the present paper is to evaluate the claims about the effects of supplements on NDI in the two replication reports.

**Methods:**

Examination of internal consistency (outcomes of statistical tests versus reported data); comparison of variability of NDI across individuals in these two reports with variability in other trials; estimation of the probability of achieving the reported close agreement with the original finding.

**Results:**

The standard deviations of NDI and levels of statistical significance reported are profoundly inconsistent. The reported standard deviations of NDI are consistently below what other studies have found. The reported percent reductions in NDI agree too closely with the original study.

**Conclusion:**

The claims of reduced NDI in the two replication reports should be questioned, which also adds to concerns about the 1992 study. It follows that there is currently no trustworthy evidence from randomized placebo-controlled clinical trials that favors the use of vitamin-mineral supplements to reduce infection in the elderly.

## Background

About 40% of the elderly in the United States take multivitamin-mineral supplements [1], and their use is increasing [2]. Studies of the clinical outcomes of such use therefore have important health and economic implications, especially as the outcomes are expected to depend on supplement constituent dosages. As shown in a recent meta-analysis [3] by El-Kadiki and Sutton, studies that examine the clinical effects of such supplements on the health of the elderly have had sharply conflicting results. The studies they considered were randomized placebo-controlled trials that examined the effects of multivitamin-mineral supplements on infection in the elderly. In most studies the participants were community-dwelling. Five of the trials showed either negative or nonsignificantly positive effects, as did an additional trial of the same type [4], published after their analysis. However, dramatic benefits were reported in the three remaining studies [5-7]. In each of these positive studies, which lasted a full year, the number of days of infection (NDI) in the supplement group was reported to be approximately half of the NDI in the placebo group. Averaged over members of the supplement groups, this indicates that the treatment reduced their NDI by about twenty days during the treatment year. Especially given the growing proportion of elderly in our population, the increasing age of retirement, and the ballooning costs of health care, such dramatic benefits would have enormous economic and quality-of-life implications.

El-Kadiki and Sutton [3] considered several hypotheses that might explain the conflict among the studies they reviewed, including differences in supplement constituents. However, the meta-analysis did not take into account the fact that the supplements in the three positive studies had identical constituents and amounts, which differed from those in the remaining studies. This identity increases the plausibility of the hypothesis that differences in supplement constituents are responsible for the conflict. An alternative possibility is that the claims in the positive reports are not to be trusted. Our purpose in the present paper is to comment briefly on the validity of the first of the positive reports [5], and to evaluate the other two [6,7], which can be regarded as reporting successful attempts to replicate the first. The possibility that trials such as these may influence decisions about required dietary allowances increases the importance of assessing their validity.

Results of the first of the positive trials were described in two papers, one [5] in 1992 in *The Lancet *("Report A"), reporting the dramatic immunological effects described above, the other [8] in 2001 in *Nutrition*, reporting greatly improved cognitive functions. These papers have been influential: they have together been cited more than 300 times; they were described in *The New York Times *[9]; and they apparently led to the founding of a company [10] to sell the supplement, for which the author holds a patent [5,10,11]. However, we and others have questioned these papers [12-16]. Problems we noted [13] in Report A [5] include inequalities in an impossible direction for each of six pairs of p values, SDs of NDI given in the text for both treatment and placebo groups that are about three times smaller than the values implied by histograms of NDI for the two groups (but about twice as large as the implied SEs), and the claim that all 96 individuals who were asked agreed to participate. Another suspicious feature, discovered recently, is that the number of observations in each of the two histograms differs from the numbers of participants in the two groups said to have completed the trial, too few in one case, too many in the other. The more numerous difficulties associated with the 2001 paper [8] were deemed sufficiently serious by the editor of *Nutrition *that he retracted it [17]. These difficulties in the 2001 paper add to our concern about Report A [5], and underline the importance of assessing the two replication studies.

The two papers reporting these replication studies ("Report B" [6] and "Report C" [7]) appeared together in the January-February 2002 issue of *Nutrition Research*. Along with 12 others of the 19 papers in that issue of the journal, these two papers were accepted within one day of being received, leaving little time for peer review. In what follows we point out three problems with the findings in both papers, problems of a similar kind.

### First problem: means and variability measures inconsistent with p values

The 1992 study and the two replications measured effects of the supplement by comparing supplement and placebo groups. Information about NDI is presented in table 4 of Report B [6] for three different time periods during the study: the first six months, the second six months, and all twelve months. Our table 1 presents those data. If we assume that the means and standard deviations (SDs) in this table are correct, and we use one tailed t tests, then, for the three tests that correspond to the three columns of the table, we get the first row of computed p values. Thus, if the means and SDs in table 4 of Report B are correct, the findings are much stronger than the reported p values would indicate – indeed, so strong as to be unbelievable. If the reported p values are correct, then we must question the means, the SDs, or both.

**Table 1:** Mean number of days of infection (mNDI) and reported versus computed significance levels from Report B [6]

	First 6 months	Second 6 months	All 12 months
Placebo Group (n = 19)	12.7 ± 1.6	11.0 ± 1.2	23.7 ± 2.3
Supplement Group (n = 22)	8.5 ± 0.7	2.6 ± 0.3	11.1 ± 0.8

Reported p values	< 0.05	< 0.004	< 0.02
Computed p values			
— Assuming "SD" = SD	< 0.0000000001	< 0.00000000000001	< 0.00000000000001
— Assuming "SD" = SE	< 0.012	< 0.000001	< 0.0001

Data are shown as mean ± SD. P values are the reported significance levels for statistical tests of the differences between placebo and supplement groups.

Perhaps instead of reporting the SD in each case, as stated, what is provided in Report B is actually the standard error of the mean (SE). (The relation between these two quantities is SE = SD/n
 MathType@MTEF@5@5@+=feaafiart1ev1aaatCvAUfKttLearuWrP9MDH5MBPbIqV92AaeXatLxBI9gBaebbnrfifHhDYfgasaacH8akY=wiFfYdH8Gipec8Eeeu0xXdbba9frFj0=OqFfea0dXdd9vqai=hGuQ8kuc9pgc9s8qqaq=dirpe0xb9q8qiLsFr0=vr0=vr0dc8meaabaqaciaacaGaaeqabaqabeGadaaakeaadaGcaaqaaiabb6gaUbWcbeaaaaa@2E2A@, where n is the sample size.) When we make this assumption (which would imply that the data are far more variable), we get the second row of computed p values in table 1. These p values are also smaller than those reported, differing by as much as a factor of 4000.

Table 2 is based on table 1 of Report C [7] which includes tests of three differences between the two groups. The table also shows the results of our computations, done in the same way as for table 1. Again there are dramatic inconsistencies and again, even if we assume that what were reported as SDs were actually SEs, the p values we calculated are much smaller than those reported, in this case by as much as a factor of 2000. See Appendix 1 for additional details.

**Table 2:** Mean data and reported versus computed significance levels from Report C [7]

	Illness Episodes	Antibiotic Days	Infection Days (mNDI)
Placebo Group (n = 18)	6.5 ± 1.0	58 ± 5	29 ± 4
Supplement Group (n = 18)	4.0 ± 0.7	27 ± 4	14 ± 2

Reported p values	> 0.05	< 0.02	< 0.03
Computed p values			
— Assuming "SD" = SD	< 0.000000001	< 0.00000000000001	< 0.0000000000001
— Assuming "SD" = SE	< 0.025	< 0.00001	< 0.001

Data are shown as mean ± SD. P values are the reported significance levels for statistical tests of the differences between placebo and supplement groups.

### Second problem: standard deviations inconsistent with other studies

One of the remarkable aspects of Reports B [6] and C [7] is how closely the reported percentage effects on NDI agree with results of the earlier study [5]. (See next section.) To evaluate the likelihood of such agreement, we need to know how variable NDI is across individuals. This is one reason why it is important to determine whether the SDs provided in Reports B and C could be as small as claimed. To do so, we examined five other studies [18-22] that reported the variability of NDI over individuals as well as its mean.

**Figure 1:**
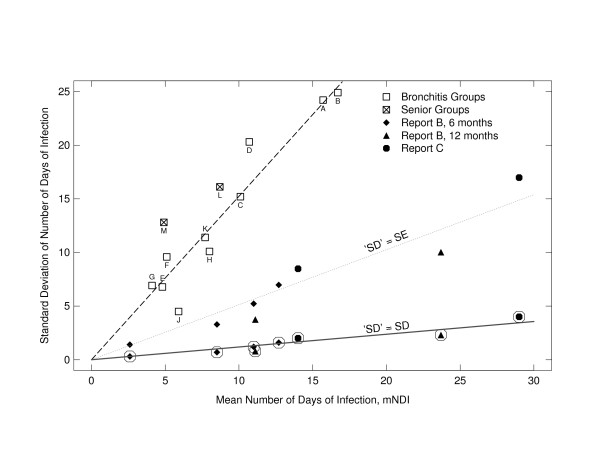
Standard deviation versus mean of the number of days of infection (NDI) for Reports B [6] and C [7] compared with those of five other studies. Filled points, representing the data reproduced in tables 1 and 2, appear twice, with circles around them (assuming that the values reported as SDs were in fact SDs), and without circles (assuming that the values reported as SDs were SEs). Also shown are linear functions fitted by least squares to the data from the bronchitis groups (top fitted line, slope = 1.59), to the circled filled points (bottom fitted line, slope = 0.13), and to the uncircled filled points (middle fitted line, slope = 0.56). See Appendix 2 for more details.

Figure 1 shows that the SDs provided in Reports B and C are an order of magnitude smaller than those reported in the comparison studies. (The ratio of slopes of the top and bottom fitted functions is 0.13/1.59 = 0.08.) If we assume that what the authors called "SDs" were actually SEs, the values in Reports B and C are still exceptionally small. (The ratio of slopes of the top and middle functions is 0.56/1.59 = 0.35.)

### Third problem: effect sizes too close to 1992 result

A third problem is the remarkably close agreement of the findings about NDI in Reports B [6] and C [7] with those in the original Report A [5]. All three papers report the mean number of days of infection (mNDI) during twelve months. These numbers, for placebo and supplement groups, are provided in table 3, along with the percent reduction in mNDI due to the supplement. The percentages in the two replication reports differ by only +0.6% and -0.3% from the percentage in the 1992 study. We show in Appendix 3 that, given plausible assumptions, the probability of such close agreement is less than 0.001, a vanishingly small probability, which indicates that the agreement of Reports B and C with Report A is too good to be true.

**Table 3:** Mean number of days of infection (mNDI) in three reports

Report	Placebo	Supplement	Percent Reduction
Report A [5]	48	23	52.1%
Report B [6]	23.7	11.1	52.7%
Report C [7]	29	14	51.8%

## Discussion

We have described three problems with the findings described in Reports B [6] and C [7], that is, three reasons to question these findings: First, the means and SDs of NDI and the levels of statistical significance that they report are profoundly inconsistent, and they remain inconsistent even if what they reported as SDs were actually standard errors of the mean. Second, the SDs of NDI that they report are more than ten times smaller than those in a set of comparison studies; if what they reported are standard errors of the mean, their SDs are about three times smaller. Third, the probability that these two studies could agree as well as they do with Report A is too small. The finding of insufficient variability has been suggested as a diagnostic for data fabrication [23,24].

A fourth problem with these findings is the fact that the two studies share these three problems. The occurrence of the same unusual features in both reports is surprising, especially as neither author acknowledged help from the other.

A fifth problem is that we have been unable to find Amrit L. Jain, the author of Report C. His institutional address ("the Medical Clinic and Nursing Home, Jaipur, India") is unverifiable; we were unable to find any trace of such a place anywhere except in his paper; the mailing address listed in his paper is a rented mailbox in Canada [15]. A letter sent to that mailing address in 2002 that asked questions about his paper was not answered. All seven papers that we found in Medline that might be authored by someone with his name date from 1978 or earlier, and are not in the fields of nutrition, aging, or infection.

A sixth problem is that in the author's reply [25] to our criticisms [13] of his Report A [5] he mentioned neither of these two replications [6,7] – an extraordinary oversight because successful replications are among the best defenses of a study against criticism.

## Conclusion

In conclusion, problems associated with Reports B [6] and C [7] of 2002, together with questions previously raised [13] about Report A of 1992 [5] favor the conclusion from Sutton and El-Kadiki's corrected meta-analysis [26] that the trustworthy evidence indicates "no benefit" from the use of multivitamin-mineral supplements for preventing infection in the elderly.

## Appendix 1: notes on tables 1 and 2

Report B [6] mentions several tests, but is not explicit about which tests were used to obtain the reported p values. Because the variances may differ between supplement and placebo groups, we computed p values by using the more conservative t test provided by Cochran [27] sometimes called the "Welch modified two-sample t test", which relaxes the assumption of equal variances. Because this was a replication study, we calculated p values for one tailed tests. (For two tailed tests these values must be doubled.) Report C [7] says nothing about which tests were used to determine significance levels. We therefore used the same procedure as for Report B.

## Appendix 2: choice of comparison studies for figure 1

The five comparison studies had group sizes ranging from 16 to 259, and covered periods of either five or six months. We found them by searching the National Library of Medicine's PubMed for articles with "days of illness" in the abstract. Because of possible distortion, we did not use studies that reported the number of days of sick leave from a job to estimate NDI. Also, we omitted variability data from Report A [5] because of the large inconsistencies between SD values stated in the text and approximate SD values derived from the histograms [13], and between the numbers of observations in the histograms and the corresponding numbers of participants.

Because the SD increases with the mean in these data, we have plotted the SDs versus the means in Figure 1. Also plotted are corresponding data from Reports B [6] and C [7]. These data are plotted twice, once assuming that what were reported as SDs are in fact SDs, and once assuming that they are SEs. Four of the five comparison studies [18-21] involved individuals with chronic bronchitis rather than normals. However, data from the fifth study [22], involving normal seniors, suggest that for a given mean, chronic bronchitis does not increase the variability of NDI.

Sources of the comparison data are as follows: Points A and B represent the number of exacerbation days during a six-month period among two groups of people with chronic bronchitis, measured by diary [18]. Points C and D represent the same, measured by interview (table 4 of [18]). Points E and F represent the number of days in bed during a five-month period among two groups of patients with chronic bronchitis complicated by severe airway obstruction (table 5 of [19]). Points G and H represent the number of days lost through illness during a six-month period among two groups of patients with chronic bronchitis (table III of [20]). Points J and K represent the number of days sick during a six-month period among two groups of patients with chronic obstructive pulmonary disease (table 2 of [21]). Points L and M represent the number of days with upper respiratory tract infection during a six month period among two groups of normal seniors (Fig. 1 of [22] and Steven M. Wood, personal communication, July 16, 2004).

Because NDI is non-negative, mNDI = 0 implies that SD(NDI) = 0; hence the linear functions fitted to the data were forced to pass through the origin. (However, even without this constraint the three fitted y values at x = 0 differ from zero by no more than one unit.)

## Appendix 3: estimation of probability of the claimed good agreement

Assuming that the trials described in Reports B [6] and C [7] were conducted and reported independently, how likely is the close agreement shown in table 3? This depends on the variability of mNDI: the greater the variability, the less likely the agreement. Unfortunately, as noted above, the different sources of information about variability (p values and SDs) within each of Reports B and C are inconsistent. Thus, to estimate the probability that the percentage in each replication report would agree as well as it did with the percentage in Report A [5], we assumed that the reported means (mNDIs) are correct, and made three different assumptions about variability to obtain estimates of the variances of the mNDIs. Because the assumption that the reported variability measures were in fact SDs led to the implausible significance levels shown in tables 1 and 2, and because of their great divergence from SDs in other studies (Fig. 1), we have not considered this possibility.

According to Assumption 1, what were called "SDs" in the replication reports were actually correct values for the SEs. The variance of mNDI, var(mNDI), is SE^2^.

According to Assumption 2, the relation between SD and mean is given by the top fitted line in Figure 1: SD = 1.59 × Mean, the line fitted to the data from the bronchitis groups. (This seems more plausible than Assumption 1.) Thus we used this equation with the mNDI values reported in Reports B and C to estimate the SDs, and from these and the sample sizes, var(mNDI).

According to Assumption 3, the reported significance levels are correct. However, whereas we need exact p values, we are given only upper bounds (p < 0.03 for Report B, p < 0.02 for Report C). We therefore used the next smaller p value (p = 0.02 for Report B, p = 0.01 for Report C), biasing the result towards higher probabilities of close agreement. Assuming that the p values were obtained from one tailed t tests, this enabled us to estimate the SEs, and from these, the values of var(mNDI). We obtained these SE estimates using three different methods, and report results from the method that produced the highest probabilities of agreement. The three methods were: (a) Conventional t test, assuming that the true SDs differ from those reported by a common factor; (b) Conventional t test, assuming that the true SDs are equal; (c) Welch modified two-sample t test, assuming that the true SDs differ from those reported by a common factor. Because of the bias mentioned above, the estimated probabilities of close agreement based on Assumption 3 are upper bounds.

Estimation of the probability also requires us to assume the form of the distribution of mNDI; we made our estimates assuming both Gaussian and gamma distributions consistent with the reported means and with the inferred values of of var(mNDI). For each of the two replication studies and each assumption, the desired probability was estimated as follows. Given the mean, inferred variance, and assumed distributional form of mNDI for the supplement and placebo groups of subjects in that study, we generated a sample of 1,000,000 pairs of mNDI values (mNDI_s _for the supplement group and mNDI_p _for the placebo group). Next, we obtained the ratio of the two numbers in each pair, (mNDI_s_/mNDI_p_). We then determined what proportion of these values were at least as close to the original finding as the observed value.

The probability that both of two independent replication studies would agree at least as well as they did with the original is the product of their separate probabilities. It is therefore the product of these two proportions, one for each replication study, that gives us the estimated probability. Under Assumption 1, the estimated probabilities for Gaussian and gamma distributions are 0.00203 and 0.00204, respectively; under Assumption 2 they are both 0.00019; and under Assumption 3 the upper bounds are 0.00066 and 0.00065, respectively.

Even under Assumption 1, which is implausible given the variability of NDI in other studies shown in Figure 1, the means and variabilities of these two sets of data make the observed closeness of agreement extremely unlikely. Under both of the other assumptions the chance of such good agreement is vanishingly small: too good to be true. These results also show that our conclusion is insensitive to the choice of distribution.

## Competing interests

We are the authors of a letter [15] in *Nutrition *criticizing the 2001 paper [8] on cognitive effects, and co-authors of a letter [13] in *The Lancet *criticizing Report A [5] based on the same trial.

## Authors' contributions

Both authors are responsible for the reasoning in the paper. SS carried out the computations and wrote the first draft. SR obtained the data for the mean and SD of NDI from the five other studies, and provided editorial comments. Both authors read and approved the final manuscript.
